# Parental Mentalizing during Middle Childhood: How Is the Adoption of a Reflective Stance Associated with Child’s Psychological Outcomes?

**DOI:** 10.3390/ijerph19106205

**Published:** 2022-05-19

**Authors:** Simone Charpentier Mora, Chiara Bastianoni, Nina Koren-Karie, Donatella Cavanna, Marta Tironi, Fabiola Bizzi

**Affiliations:** 1 Department of Educational Sciences, University of Genova, 16128 Genoa, Italy; simone.charpentiermora@edu.unige.it (S.C.M.); chiara.basti@gmail.com (C.B.); donatella.cavanna@unige.it (D.C.); marta.tironi@edu.unige.it (M.T.); 2 The Center for the Study of Child Development, School of Social Work, University of Haifa, Haifa 3498838, Israel; nkoren@research.haifa.ac.il

**Keywords:** parental mentalizing, parental insightfulness, Parental Reflective Functioning, psychological symptoms, social–emotional competencies, middle childhood

## Abstract

This exploratory cross-sectional study attempts to understand the mechanisms underlying the role of parental mentalizing in a child’s psychological functioning during middle childhood by using Parental Reflective Functioning (PRF) and Parental Insightfulness (PI) constructs. The main aims are to examine the role of PI and PRF as processes capable of influencing a child’s psychological functioning in terms of emotional–behavioral difficulties and social–emotional competencies. Eighty-six community parents (48 mothers, 38 fathers) and their 50 children in middle childhood (Mage = 10.10, SD = 1.13) participated in this study, recruited through a non-probabilistic sampling. The following measures were used to assess the aims of this study: Insightfulness Assessment, Parental Reflective Functioning Questionnaire, Child Behavior Checklist (CBCL) and Devereux Student Strengths Assessment (DESSA) questionnaires. Results showed that parental mentalizing was found to be significantly associated with both child’s internalizing and externalizing symptoms and social–emotional competencies as reported by parents through the CBCL and DESSA questionnaires. This study may offer a contribution to the study of parental mentalizing during middle childhood, supporting the hypothesis that both parents’ ability to understand their child’s mental states could affect the child’s psychological functioning. Clinical and theoretical implications are geared toward a family-based view with a specific focus on the importance of fostering in both parents a positive attitude toward mentalizing processes.

## 1. Introduction

Middle childhood represents for the child and his family a period of important changes that occur on several levels. At the individual level, we witness the maturational evolution of a child’s cognitive and emotional processes parallel to pubertal growth. These processes also include significant development of the child’s mentalization and social cognition skills [1,2]. Concurrently, at a social level, there is an expansion of the relevance that the extra-familiar context assumes for child development. Relationships with peers, primarily grounded within the school context, become a significant source of exploration and enhancement of social–emotional competencies [3].

Although such important changes make this stage of child’s life of great interest, studies related to attachment theory that have addressed middle childhood are relatively lacking [4,5,6]. Furthermore, although there has been an increase in research in recent years, several gaps related to the study of parental mentalizing, the father’s role, and the link between parenting variables and a child’s psychological and psychopathological outcomes still remain (see [7] for a review).

Following the framework outlined by attachment theory, this exploratory investigation moves its steps in light of the various studies (e.g., [8,9,10,11]) that showed links between a child’s socio-emotional and cognitive development and parental mentalizing intended as the foundation on which parental behavior toward their child is built. Parental mentalizing is thus presumed to shape a child’s expectations regarding parental emotional availability, and also how the child felt that his feelings, thoughts and wishes were understood and accepted by his parent.

### 1.1. Parental Mentalizing: Theory and Assessment

Mentalizing construct, originally operationalized as Reflective Functioning (RF) by Peter Fonagy and colleagues [12], has gained wide space over the last thirty years. Early studies thus focused on the predictive value of the quality of parents’ mentalizing with respect to their child’s psychological outcomes [12,13], which are closely related to the quality of family relationships [7].

It is in this line of research that David Oppenheim and Nina Koren-Karie [14] and Arietta Slade [15] proposed respectively the two concepts of Parental Insightfulness (PI) and Parental Reflective Functioning (PRF). The construct of PRF—i.e., a parent’s ability to reflect upon their child’s inner mental world [15]—expands the goal of measuring RF by exploring it directly within the parent–child relationship and investigating parents’ ability to understand their own and their child’s internal experience in terms of intentional mental states. In this regard, a modified version of the Reflective Functioning Scale [16] was implemented and applied to the Parental Development Interview (PDI [17]) an interview specifically designed to explore the parental ability to reflect on the ongoing parent–child relationship. Unlike the original Reflective Functioning Scale (RFS [18]), which uses transcripts related to past relational experiences from the Adult Attachment Interview [19], Slade’s Reflective Functioning Scale uses current material—that is obtained from PDI transcripts—related to dyadic interactions occurring in the present moment. Over the past few years, a less time-consuming measure for assessing mentalizing has been developed: the Parental Reflective Functioning Questionnaire (PRFQ [20]). PRFQ is a self-report questionnaire that measures key dimensions of parental mentalizing including interest and curiosity about their child’s mental states, the parent’s pre-mentalizing modes of thinking and certainty about the child’s mental states.

The construct of PI, similarly to that of PRF, indicates a parent’s ability to think about the processes underlying their child’s behavior in an accepting, complex and open manner, taking into account the child’s perspective [21]. Differently from the PDI, Nina Koren-Karie and David Oppenheim have developed a video-replay assessment procedure—the Insightfulness Assessment procedure (IA [22,23])—that explores parental mentalizing employing several videotaped segments of the parent–child dyadic interaction that are viewed and then commented on by the parent along with the interviewer. Thus, PI could be understood as a concept contiguous with that of PRF although it should be distinguished from it at least procedurally, in that to be measured it requires the parent to observe a videotaped interaction with their child. PI thus refers to the parent’s ability to be able to convey an image of the child that is emotionally complex and open to change, understanding the child’s mental states in the here and now of the relationship with the child [23]. In doing so, the main aim of its evaluation focuses on the parent’s ability to think about the motives that underlie the child’s behavior while linking these thoughts to the child’s behavior [24]. It is based on Ainsworth’s description of the sensitive mother as “seeing things from the child’s point of view” [25] and is assessed using a video-replay method in which parents are interviewed about “what went on in your child’s head” after watching several video clips of the child interacting with them. Thus, parents are required to engage in a “dialogue” between what they know about their child based on their shared history and the specific behaviors and reactions of the child as captured on video. A task requiring the parent to understand, and especially to accept the child as a separate person with their own thoughts, emotions, needs and desires different from those of the parent and sometimes contradictory to their own goals [21]. Moreover, by virtue of its procedure for mentalizing evaluation, the Insightfulness Assessment procedure could constitute a potential intervention model able to increase a parental mentalizing stance similar to video-feedback interventions and could also be used prior to mentalization-based parenting programs to target therapeutic goals according to the specific mentalistic deficits (e.g., [26]).

### 1.2. Parental Mentalizing and Child’s Psychological Outcomes: Which Role for Parental Reflective Functioning and Parental Insightfulness?

The construct of parental mentalizing, which has been identified and measured in this study throughout the two constructs of Parental Reflective Functioning (PRF [15]) and Parental Insightfulness (PI [14]), allows us to place attachment theory within a broader and more complex context defined by parental ability to understand a child’s behaviors on the basis of intentional mental states. Within this framework, many studies have shown the association between parental mentalizing and a child’s psychological and psychopathological outcomes, suggesting an important role played by familial relationships (for a review [7,27]).

As regards PRF construct, the studies listed below mostly used an interview-based assessment of mentalizing (e.g., RFS [16,18]) while a few studies [20,28,29,30,31] used the newly developed Parental Reflective Functioning Questionnaire [20] which is easier and more convenient to operate but prone to the tradeoffs of self-report questionnaires. These studies have highlighted the role of PRF in regard to a child’s psychopathological symptoms by showing a negative association between good parental mentalizing and child’s internalizing and externalizing problems [28,30,31,32,33,34,35,36,37], and better socio-emotional competencies and fewer socio-emotional problems for the child [31,38]. Other studies have shown a negative association between parental mentalizing and a child’s anxiety levels [39] and a positive relationship with a child’s mentalizing [40,41,42,43]. Finally, additional studies have shown significant associations between parental mentalizing and child attachment security [20,44,45,46,47,48,49,50] and higher child emotional regulation competencies [51]. Finally, other studies suggested a protective role of parental mentalizing, some of which have shown a moderating role of maternal PRF within the relationship between difficult temperament and a child’s behavioral difficulties [52] and between emotional distress and a child’s coping strategies during a stress-task [29].

Moreover, concerning PI, several studies have shown its relationship to a child’s attachment security [53,54,55,56,57], lower child externalizing symptoms [11], lower child externalizing and internalizing symptoms [26], higher child cognitive abilities [58], and higher child responsiveness and involvement during a mother–child dyadic interaction [59]. Finally, some studies showed a protective role of PI within the relationship between a child’s exposure to violent behaviors and the presence of internalizing and externalizing symptoms and negative affectivity [60].

Overall, these studies have outlined the relationship between parental mentalizing and a child’s psychological outcomes, stressing the key role played by mentalistic representations that parents have of their children and the influence that these representations may have on a child’s psychological trajectories. However, most of the research has mainly focused on early childhood and on the mother’s role, leaving aside later developmental stages such as middle childhood and adolescence except for a few studies [33,34,39,40,41,42,43,44,61,62,63]—together with the father’s mentalizing role. Following this direction, some studies addressed the relationship between paternal PRF and a child’s psychological outcomes, showing associations with Attention-Deficit/Hyperactivity Disorder [64], child social–emotional competencies [31,65], child internalizing and externalizing symptoms [61], and child anger propensity [66], while other studies did not find significant associations [39,63,67,68,69,70].

Furthermore, most of the studies focused on children’s psychopathological outcomes—e.g., internalizing and externalizing symptomatology—while a gap in studies of other children’s psychological dimensions such as social–emotional competencies (e.g., [31,38]) and prosocial behaviors still remains. The inclusion of parental mentalizing as an explanatory variable is still limited to a few studies. For example, Nijssens et al. [65] highlighted the role of both parents’ PRF as a mediator within the relationship between parental attachment dimensions and a child’s social–emotional competencies in families with children aged 8 to 13 months. Gordo et al. [71] showed the indirect effect of both parents’ PRF on child’s social–emotional adjustment by mediating the role of perceived parental competence in families with children aged 2 to 36 months. Finally, León and Olhaberry [69] showed, in families with children aged 12 to 38 months, the absence of a direct relation between both parents’ PRF and the child’s social–emotional behavior while the indirect effect of maternal PRF alone on the child’s social–emotional behavior was shown throughout the mediational role of the quality of triadic family interaction.

Regarding the role of PI, one study [72] investigated the role of both paternal and maternal Insightfulness in relation to a child’s involvement during a triadic family interaction but showed no significant effects. However, although there have been no studies that have explored the link with child’s social–emotional competencies, we may hypothesize that higher levels of PI could be associated with higher levels of child’s social–emotional competencies [24]. Indeed, as Stein [73] (p. 309) suggests, “the most obvious place to begin the search for mechanisms of resilience is the family” and studies that explore the relational competencies within the family unit are necessary because of their incidence in the area of primary prevention, also opening the way to possible interventions focused not only on reducing psychopathological difficulties but also and especially on promoting the psychological resources of child and his family [74,75].

### 1.3. The Current Study

Based on the analysis of the literature and having shown the lack of research addressing parental mentalizing—both PRF and PI—especially in reference to middle childhood, the current study aimed to explore the relationship between parental mentalizing and children’s emotional–behavioral difficulties and social–emotional competencies in a community sample composed of families with their children during this specific developmental stage.

Specific aims include:

(1) exploring the relationship between parental mentalizing—measured throughout a multi-method approach consisting in the use of the Insightfulness Assessment procedure (IA) and the Parental Reflective Functioning Questionnaire (PRFQ)—and a child’s psychological symptoms—measured in terms of internalizing and externalizing symptomatology—;
(2) exploring the relationship between parental mentalizing and a child’s social–emotional competencies.

Starting from these aims, we hypothesized that higher levels of parental mentalizing would be associated with: (1) lower levels of child’s internalizing and externalizing symptoms; and (2) higher levels of child’s social–emotional competencies.

## 2. Materials and Methods

### 2.1. Participants

The participants in the present study are part of a larger project entitled “From parental mentalizing to the child’s psychological and psychopathological outcomes during middle childhood” which involved Italian families with children aged 8 to 12 years after a non-probabilistic convenience sampling throughout different schools located in a large city in the north of Italy.

A total of 87 parents (N = 49 mothers, N = 38 fathers) and their 50 children (Mage = 10.10, SD = 1.13; 60% males) were included in this study.

The inclusion criteria were: (1) age of the children between 8 and 12 years; (2) absence of child psychopathology (i.e., parents reported that their children had no psychiatric diagnosis. In addition, participating children did not exceed clinical cut-off—total problem score t ≤ 65—for emotional–behavioral difficulties. Child’s total symptoms on Child Behavior Checklist/6-18 were: M = 51.3, SD = 10.00 for mother-report and M = 49.2, SD = 8.87 for father-report); (3) sufficient Italian language comprehension for both children and parents.

### 2.2. Procedure

The present study was conducted in accordance with the ethical standards of the American Psychological Association [76] and was approved by the Research Ethics Committee of the Department of Educational Sciences (University of Genova, Italy) (protocol N. 023/2018).

All participating families signed a written informed consent. Questionnaire and interviews administration has been split into two meetings with each family in order to avoid participants’ fatigue. In the first meeting parents were asked to fill out a questionnaire about socio-anagraphic characteristics, parent-report questionnaires about child’s psychological functioning—i.e., Child Behavior Checklist/6-18 [77] and Devereux Student Strengths Assessment [78]—and Parental Reflective Functioning Questionnaire [20]. Three video recordings about parent–child interactions were subsequently made for each parent. During the second meeting, the Insightfulness Assessment interview was administered to assess PI.

### 2.3. Measures

The tools used to assess the study’s aims were:

- A socio-demographic questionnaire for collecting family data (age, sex, parents’ years of schooling, and family’s socio-economic status—high  >  36,000 €/y, moderate to low ≤ 36,000 €/y—).
- Parental Reflective Functioning Questionnaire (PRFQ [20,79]) for assessing PRF: PRFQ is a self-report questionnaire with a seven-point Likert scale. It consists of 18 questions divided into three domains: Pre-Mentalizing Modes (PM, 6 items: e.g., “The only time I’m certain my child loves me is when he or she is smiling at me”), which measures parent’s difficulty in accurately understanding child’s mental states even to the extent of having malicious attributions and an inability to understand the child’s inner world; Interest and Curiosity about Mental States (IC, 6 items: e.g., “I like to think about the reasons behind the way my child behaves and feels”), which measures the parent’s ability to show a curious stance around their child’s mental states, and Certainty about Mental States (CMS, 6 items: e.g., “I can completely read my child’s mind”), which measures parent’s overconfidence in thinking what the child may think or feel (i.e., hypermentalizing) but, on the other side, also measure a lack of access to child’s mental states (i.e., hypomentalizing). In the current study, PRFQ showed good internal consistency for all domains (CMS: mothers, McDonald’s ω = 0.85; fathers, McDonald’s ω = 0.76; IC: mothers, McDonald’s ω = 0.79; fathers, McDonald’s ω = 0.69; PM: mothers, McDonald’s ω = 0.62; fathers, McDonald’s ω = 0.71).
- Insightfulness Assessment (IA [14]) for assessing PI: IA is a video-replay based procedure in which parents are asked to engage with the child in three dyadic interactions that are video-recorded. These interactions included a competitive game (i.e., Jenga Tower), a cooperative game (i.e., building a 3D puzzle) and a free play. Parents are subsequently interviewed by showing a 2-min video segment from each interaction and by asking about what went through their child’s mind (i.e., how the child feels and thinks), whether child’s behavior during the segment was typical and how the parent felt watching the segment. The interview also requires parents to support their answers with examples from child’s observation (e.g., “what do you think the child felt in this specific segment?”) and everyday life. The content of that interview is audio-recorded, transcribed and coded throughout ten dimensional subscales by the first author trained by Nina Koren-Karie with inter-rater reliability (ICC (2,2)) calculated on 20% of cases (presented in parentheses): Insight into child’s motives (0.83), Acceptance of the child (0.75), Openness/flexibility of thought (0.62), Complexity in description of child (0.77), Maintenance of focus on child (0.62), Anger (0.67), Separateness from child (0.74), Concern (0.78), Richness of description of child (0.65) and Coherence of thought (0.88). Each subscale provides a score from 1 to 9 and then the scores may be converted for both nominal categories (Insightful, Non-Insightful) and a dimensional composite score. Following earlier studies using IA (e.g., [80]) a dimensional composite PI score was preferred over nominal categories to increase statistical power of our study. Thus, individual subscales were averaged to obtain an Insightfulness Total Score (ITS) which showed a good internal consistency (mothers, McDonald’s ω = 0.93; fathers, McDonald’s ω = 0.98) and a good inter-rater reliability (ICC (2, 2) = 0.84).
- Child Behavior Checklist/6-18 (CBCL [77,81]) for assessing a child’s emotional–behavioral difficulties: CBCL is a parent-report questionnaire with a three-point Likert scale. It consists of 112 questions divided into three composite syndromic scales—Internalizing Symptoms, Externalizing Symptoms, Total Symptoms—and six DSM-oriented scales. The three syndromic scales were used in the current study showing a good internal consistency: Internalizing Symptoms (mothers, McDonald’s ω = 0.87; fathers, McDonald’s ω = 0.82), Externalizing Symptoms (mothers, McDonald’s ω = 0.81; fathers, McDonald’s ω = 0.77) and Total Symptoms (mothers, McDonald’s ω = 0.88; fathers, McDonald’s ω = 0.89).
- Devereux Student Strengths Assessment (DESSA [78,82]) for assessing a child’s social–emotional competencies: DESSA is a parent report questionnaire with a five-point Likert scale. It consists of 72 questions divided into eight individual scales—Self-awareness, Social Awareness, Self-Management, Goal-Directed Behavior, Relationship Skills, Personal Responsibility, Decision Making and Optimistic Thinking—and a composite scale—Social Emotional Composite (SEC)—. In the current study, SEC was only used showing a good internal consistency (mothers, McDonald’s ω = 0.91; fathers, McDonald’s ω = 0.93).

### 2.4. Statistical Analyses

All statistical analyses were performed using jamovi statistical package [83]. Descriptive statistics were run using means and standard deviations for continuous variables while frequencies and percentages for categorical variables.

Firstly, we assessed associations between study’s variables and sociodemographic variables, in order to include potential covariates in subsequent analyses.

Secondly, Rho Spearman’s and Bravais–Pearson correlation coefficients were used to assess the association between parental mentalizing, the child’s emotional-behavior difficulties and the child’s social–emotional competencies. Hierarchical regression models were subsequently performed to estimate the possible predictive role of parental mentalizing on a child’s psychological outcomes. Statistical results were considered significant with a *p* value < 0.05. There was no evidence of multicollinearity problems (tolerance values > 0.05 and VIF values < 2 for all models) and study variables were normally distributed according to the Shapiro–Wilk test.

## 3. Results

### 3.1. Preliminary Analyses and Descriptive Statistics

Eighty-seven parents and their 50 children participated in the study. Table 1 shows socio-demographic characteristics of the sample. Descriptive statistics of parental mentalizing, the child’s psychological symptoms and the child’s social–emotional competencies are shown in Table 2. Preliminary analyses were conducted in order to explore the link between the study variables and sociodemographic variables. Firstly, correlation analyses showed no relationship between child, mother and father’s age, and parental years of schooling, except for a significant association between mother’s age and mother-report Internalizing Symptoms (r = 0.48, *p* < 0.01).

Secondly, we conducted 2 × 2 MANOVAs including child’s sex and family’s socio-economic status as independent variables and CBCL scales (Internalizing Symptoms, Externalizing Symptoms, Total Symptoms) as dependent variables.

Finally, we conducted a 2 × 2 ANOVA including child’s sex and family’s socio-economic status as independent variables and Social Emotional Composite scale from DESSA as a dependent variable.

Analyses showed no relationship between the child’s sex, the family’s socio-economic status, and the study’s variables (*p* values from 0.09 to 0.90).

In conclusion, the mother’s age was only included as a covariate in subsequent analyses.

**Table 1:** Socio-demographic characteristics of the sample.

	N	Mean (Standard Deviation)
Mother’s age	49	45.20 (5.73)
Father’s age	37	46.40 (5.40)
Child’s age	50	10.10 (1.13)
Mother’s years of schooling	49	16.20 (4.07)
Father’s years of schooling	37	15.30 (4.49)
	**N = 49**	**Frequency (%)**
Family’s socio-economic status	≤36,000 €/year	21 (42.9)
	>36,000 €/year	28 (57.1)

**Table 2:** Descriptive statistics of parental mentalizing, child’s psychological symptoms and child’s social–emotional competencies.

		Mean	Standard Deviation
ITS	Mother	5.29	0.82
	Father	5.50	0.94
PRFQ-PM	Mother	1.71	0.57
	Father	1.57	0.46
PRFQ-IC	Mother	5.77	0.97
	Father	5.56	0.81
PRFQ-CMS	Mother	3.98	1.23
	Father	4.27	0.99
CBCL-I	Mother	53.9	11.3
	Father	51.8	10.4
CBCL-E	Mother	50.2	8.11
	Father	48.5	7.34
CBCL-T	Mother	51.3	10.0
	Father	49.2	8.87
SEC	Mother	49.6	7.13
	Father	50.8	7.98

Note. ITS = Insightfulness Total Score; PRFQ-PM = Pre-Mentalizing Subscale; PRFQ-IC = Interest and Curiosity Subscale; PRFQ-CMS = Certainty about Mental States Subscale; CBCL-I = Internalizing Symptoms; CBCL-E = Externalizing Symptoms; CBCL-T = Total Symptoms; SEC = Social Emotional Composite.

### 3.2. Association between Parental Mentalizing and Child’s Psychological Symptoms

In order to explore the link between parental mentalizing and a child’s psychological symptoms, we initially conducted a series of correlation analyses between both parents’ PRFQ scales and Insightfulness Total Score and the child’s Internalizing, Externalizing and Total Symptoms (Table 3).

As Table 3 shows, analyses revealed significant associations for the mother’s Insightfulness Total Score, the mother’s Pre-Mentalizing subscale and the father’s Pre-Mentalizing subscale.

A series of linear regression models were subsequently run predicting mother- and father-report CBCL scales (Table 4).

The first three regression models have regarded the prediction of mother-report CBCL scales. The first model concerned the prediction of the child’s Internalizing Symptoms. Within this model, after entering the mother’s age in an initial step (R^2^ = 0.24, *p* = 0.001), the mother’s Insightfulness Total Score (∆R^2^ = 0.12, *p* = 0.01) and the mother’s Pre-Mentalizing subscale (∆R^2^ = 0.10, *p* = 0.01) were entered in two subsequent steps. Overall, the final model significantly explained about 45% of the variability in mother-report child’s Internalizing Symptoms: mother’s age (b = 0.94, SE = 0.27, *p* = 0.001), mother’s Insightfulness Total Score (b = −4.94, SE = 1.89, *p* = 0.01) and mother’s Pre-Mentalizing subscale (b = 6.97, SE = 2.72, *p* = 0.01) are significant predictors of the child’s internalizing symptomatology although once the mother’s Pre-Mentalizing subscale was also included, the link between the mother’s Insightfulness Total Score and the child’s Internalizing Symptoms has become non-significant.

The second model concerned the prediction of the child’s Externalizing Symptoms. Within this model, the mother’s Pre-Mentalizing subscale (R^2^ = 0.12, *p* = 0.02) was only entered Overall, the final model significantly explains about 12% of the variability in the mother-report child’s Externalizing Symptoms: the mother’s Pre-Mentalizing subscale (b = 4.90, SE = 1.96, *p* = 0.02) was a significant predictor of the mother-report child’s externalizing symptomatology.

The third model concerned the prediction of the child’s Total Symptoms. Within this model, the mother’s Insightfulness Total Score (R^2^ = 0.13, *p* = 0.02) and the mother’s Pre-Mentalizing subscale (∆R^2^ = 0.11, *p* = 0.03) were entered in two consecutive steps. Overall, the final model significantly explains about 24% of the variability in the mother-report child’s Total Symptoms: mother’s Insightfulness Total Score (b = −4.54, SE = 1.89, *p* = 0.02) and mother’s Pre-Mentalizing subscale (b = 6.34, SE = 2.75, *p* = 0.03) are significant predictors of the child’s total symptomatology although once the mother’s Pre-Mentalizing subscale was also included, the link between the mother’s Insightfulness Total Score and the child’s Total Symptoms has become non-significant.

The last two regression models have regarded the prediction of the father-report CBCL scales. The first model concerned the prediction of the child’s Internalizing Symptoms. Within this model, the father’s Pre-Mentalizing subscale was included in a single step (R^2^ = 0.14, *p* = 0.02), which turns out to be a significant predictor explaining about 14% of the variability in the father-report child’s Internalizing Symptoms.

The second model concerned the prediction of the child’s Total Symptoms. Within this model, the mother’s Pre-Mentalizing subscale was included in a single step (R^2^ = 0.12, *p* = 0.04) which turns out to be a significant predictor explaining about 12% of the variability in the father-report child’s Total Symptoms.

**Table 4:** Hierarchical multiple regression models predicting child’s psychological symptoms.

	**Overall Model CBCL-I (Mother): F (3, 36) = 10.00, R^2^ = 0.45, *p* < 0.001**			
	b	SE	t	CI
Model 1				
Mother’s age	0.94	0.27	3.43 ***	[0.38, 1.49]
Model 2				
Mother’s age	0.92	0.26	3.62 ***	[0.41, 1.44]
ITS (mother)	−4.94	1.89	−2.61 **	[−8.77, −1.11]
Model 3				
Mother’s age	1.00	0.24	4.16 ***	[0.51, 1.48]
ITS (mother)	−2.93	1.93	−1.52	[−6.84, 0.98]
PRFQ-PM (mother)	6.97	2.72	2.56 **	[1.46, 12.48]
	**Overall Model CBCL-E (Mother): F (1, 46) = 6.24, R^2^ = 0.12, *p* < 0.05**			
	b	SE	t	CI
Model 1				
PRFQ-PM (mother)	4.90	1.96	2.50 *	[0.95, 8.85]
	**Overall Model CBCL-T (Mother): F (2, 37) = 5.85, R^2^ = 0.24, *p* < 0.01**			
	b	SE	t	CI
Model 1				
ITS (mother)	−4.54	1.89	−2.39 *	[−8.37, −0.70]
Model 2				
ITS (mother)	−2.72	1.96	−1.39	[−6.69, 1.25]
PRFQ-PM (mother)	6.34	2.75	2.30 *	[0.76, 11.91]
	**Overall Model CBCL-I (Father): F (1, 34) = 5.47, R^2^ = 0.14, *p* < 0.05**			
	b	SE	t	CI
Model 1				
PRFQ-PM (father)	8.34	3.57	2.34 *	[1.10, 15.59]
	**Overall Model CBCL-T (Father): F (1, 34) = 4.56, R^2^ = 0.12, *p* < 0.05**			
	b	SE	t	CI
Model 1				
PRFQ-PM (mother)	7.30	3.42	2.14 *	[0.35, 14.25]

Note. CBCL-I = Internalizing Symptoms; CBCL-E = Externalizing Symptoms; CBCL-T = Total Symptoms; ITS = Insightfulness Total Score; PRFQ-PM = Pre-Mentalizing Subscale; b = unstandardized beta; SE = Standard Error; CI = Confidence Interval 95%; * *p* < 0.05, ** *p* < 0.01, *** *p* < 0.001.

### 3.3. Association between Parental Mentalizing and Child’s Social–Emotional Competencies

In order to explore the link between parental mentalizing and a child’s social–emotional competencies, we initially conducted a series of correlation analyses between both parents’ PRFQ scales and Insightfulness Total Score and the child’s Social Emotional Composite (Table 3).

As Table 3 shows, analyses revealed significant associations for the mother’s Insightfulness Total Score, the mother’s Pre-Mentalizing subscale, the mother’s CMS subscale, the father’s CMS subscale and the father’s IC subscale.

A series of two linear regression models were subsequently run, predicting the mother- and father-report DESSA scale (Table 5).

The first regression model concerned the prediction of the mother-report Social Emotional Composite. Within this model, the mother’s Pre-Mentalizing subscale and the mother’s CMS subscale (R^2^ = 0.38, *p* < 0.001), and the father’s CMS subscale (∆R^2^ = 0.13, *p* = 0.005) were entered in two subsequent steps. Overall, the final model significantly explains about 51% of the variability in the mother-report child’s Social Emotional Composite: the mother’s Pre-Mentalizing subscale (b = −9.09, SE = 2.35, *p* < 0.001), mother’s CSM subscale (b = 1.73, SE = 0.85, *p* = 0.05) and father’s CMS subscale (b = −2. 72, SE = 0.92, *p* = 0.006) are significant predictors of the child’s social–emotional competencies.

The second regression model concerned the prediction of the father-report Social Emotional Composite. Within this model, the mother’s Insightfulness Total Score Mother (R^2^ = 0.16, *p* = 0.03), mother’s Pre-Mentalizing subscale (∆R^2^ = 0.07, *p* = 0.14), and father’s IC subscale (∆R^2^ = 0.28, *p* < 0.001) were entered in three subsequent steps. Overall, the final model significantly explains about 51% of the variability in the father-report child’s Social Emotional Composite: the mother’s Insightfulness Total Score (b = 3.64, SE = 1.57, *p* = 0.03) and father’s IC subscale (b = 4.98, SE = 1.26, *p* < 0.001) are significant predictors of the child’s social–emotional competencies.

**Table 5:** Hierarchical multiple regression models predicting a child’s social–emotional competencies.

	**Overall Model SEC (Mother): F (3, 34) = 11.75, R^2^ = 0.51, *p* < 0.001**			
	b	SE	t	CI
Model 1				
PRFQ-PM (mother)	−9.09	2.35	−3.87 ***	[−13.87, −4.32]
PRFQ-CMS (mother)	1.73	0.85	2.04 *	[0.01, 3.45]
Model 2				
PRFQ-PM (mother)	−9.08	2.13	−4.27 ***	[−13.40, −4.76]
PRFQ-CMS (mother)	1.62	0.77	2.11 *	[0.06, 3.18]
PRFQ-CMS (father)	−2.72	0.92	−2.96 **	[−4.58, −0.85]
	**Overall Model SEC (Father): F (3, 27) = 9.20, R^2^ = 0.51, *p* < 0.001**			
	b	SE	t	CI
Model 1				
ITS (mother)	3.64	1.57	2.32 *	[0.43, 6.85]
Model 2				
ITS (mother)	2.77	1.64	1.69	[−0.58, 6.13]
PRFQ-PM (mother)	−4.92	3.21	−1.53	[−11.50, 1.66]
Model 3				
ITS (mother)	3.94	1.36	2.89 **	[1.15, 6.73]
PRFQ-PM (mother)	−3.74	2.62	−1.43	[−9.13, 1.64]
PRFQ-IC (father)	4.98	1.26	3.94 ***	[2.39, 7.57]

Note. SEC = Social Emotional Composite; ITS = Insightfulness Total Score; PRFQ-PM = Pre-Mentalizing Subscale; PRFQ-CMS = Certainty about Mental States Subscale; PRFQ-IC = Interest and Curiosity Subscale; b = unstandardized beta; SE = Standard Error; CI = Confidence Interval 95%; * *p* < 0.05, ** *p* < 0.01, *** *p* < 0.001.

## 4. Discussion

The present exploratory study aimed to explore the link between parental mentalizing and children’s psychological functioning in a community sample during middle childhood. More specifically, our aims were pursued throughout a multi-method approach assessing parental mentalizing by using the Insightfulness Assessment procedure (IA [14]) and Parental Reflective Functioning Questionnaire (PRFQ [20]) in order to get a more differentiated image about the role of this parental competence on the child’s psychological functioning. In this regard, the two measures allowed us to explore two different configurations of parental mentalizing. PRFQ provides a self-assessment of one’s own mentalizing throughout a questionnaire divided into three dimensional scales, while IA provides a moment-by-moment measurement of mentalizing competencies stimulating the parent to reflect about his/her child’s inner world throughout the use of videotaped footages of dyadic parent-child interaction. The theoretical framework that supported our study is highlighted by several studies (e.g., [84,85,86,87]), which stressed the link between family context and a child’s psychological functioning.

On this basis, we expected to find a significant relationship between parental mentalizing and their child’s psychological functioning. In line with previous studies [28,31,32,33,34,36,37,88], our findings showed a significant role of maternal Insightfulness and mother’s and father’s pre-mentalizing modes. Going into detail, the data suggested that: (1) both maternal Insightfulness and mother’s pre-mentalizing modes have a significant—and inverse—effect on mother-report child’s internalizing symptomatology; (2) exclusively mother’s pre-mentalizing modes have a significant effect on mother-report child’s externalizing symptomatology; while (3) both maternal Insightfulness and mother’s pre-mentalizing modes have a significant—and inverse—effect on mother-report child’s total symptomatology. As regards the father-report CBCL scores, the data suggested that: (1) father’s pre-mentalizing modes have a significant effect on the child’s internalizing symptomatology; while (2) mother’s pre-mentalizing modes only have a significant effect on child’s total symptomatology. Overall, we can state that the mother’s Insightfulness—i.e., the mother’s ability to grasp the mental states underlying their child’s behaviors in a positive, accepting, and flexible manner—represents a protective factor against the child’s emotional–behavioral difficulties, whereas both parents’ pre-mentalizing modes—i.e., parents’ difficulty in accurately understanding their child’s mental states by having a flawed mentalizing ability in reading their child’s behaviors—represent risk factors where such difficulties exist.

These findings also stressed a stronger contribution of maternal mentalizing. The father’s pre-mentalizing modes only—and limited to father-report child’s internalizing symptomatology—played a significant role, suggesting that, within our sample, the role of paternal mentalizing, only in the presence of the fathers’ difficulties in keeping in mind his child’s inner world, is exclusively related to the child’s emotional–behavioral difficulties. Some explanations might be ventured in an attempt to discuss these findings, also including the specific characteristics of the father–child relationship during middle childhood. Indeed, it is possible to argue that the father, as suggested in other studies (e.g., [71]), is little involved in emotional interactions with the child, thus making paternal mentalizing less influential on a child’s emotional–behavioral difficulties except as a risk factor with regard to major mentalistic deficits, as in the case of pre-mentalizing modes. In this regard, several studies have shown a greater influence of maternal mentalizing [12,39,63,68,69,89], other studies have shown that maternal and paternal mentalizing have a similar influence [64,90] while still others have shown a greater influence of the paternal one [61,91].

However, it should be pointed out that studies have also shown mixed results about differences in levels of paternal and maternal mentalizing abilities. Several studies showed similar levels of maternal and paternal mentalization [44,69,92,93,94], while other studies [61,65,79,91,95,96,97] have shown different levels. As noted by Ruiz et al. [98], it would be worthwhile to explore not only the intensity with which parents use their mentalizing processes, but the specific thematic content of these processes and the different variables involved in their increase or deficit. Therefore, future studies are needed to explore this hypothesis in larger and more differentiated samples to obtain a clearer picture of this gender difference.

Regarding the link between parental mentalizing and children’s social–emotional competencies, our findings have shown a significant role of the mother’s pre-mentalizing modes, maternal Insightfulness, the father’s certainty about mental states and the father’s interest and curiosity about mental states. Going into detail, the data suggested that higher mother-report child’s social–emotional competencies were found to be associated with: (1) higher levels of mother’s certainty about mental states; (2) lower levels of the mother’s pre-mentalizing modes; and (3) lower levels of the father’s certainty about mental states. Secondly, higher father-report child’s social–emotional competencies were found to be associated with: (1) higher levels of maternal Insightfulness; and (2) higher levels of the father’s interest and curiosity about mental states. Despite the aforementioned literature gap in deepening these associations during middle childhood, our findings show continuity with other studies that investigated the link between parental mentalizing and a child’s social–emotional competencies (e.g., [31,38,61,65,69,71]). The results obtained thus suggest the importance of paying attention not only to the child’s psychological difficulties but also to the paths that lead to an enhancement of one’s psychological functioning such as linking mentalizing to the construct of resilience (e.g., [74]).

In an attempt to comment on the results, it would also be interesting to note that maternal and paternal certainty about mental states are inversely associated with the child’s social–emotional competence. Despite excessive certainty about mental states may indicate hypermentalizing and thus be maladaptive, in the PRFQ’s validation study, Luyten et al. [20] have shown a positive association between certainty about the mental states and features of parental emotional availability. Therefore, the average level of certainty about mental states may also be considered an adaptive parental feature. Moreover, since in our study maternal CMS values are similar to data from the Italian validation of PRFQ [79] that used a community sample, maternal certainty about mental states could be considered a variable that describes a good mentalizing functioning with positive effects on a child’s social–emotional competence. Paternal CMS, however, has offered an alternative interpretation of events by showing a negative association with a child’s social–emotional competence. This result could be understood due to the differences between the role of paternal and maternal mentalizing shown by several studies [12,39,61,63,68,69,89,91]. Therefore, future studies are needed to better comprehend the role of certainty about mental states within different child developmental stages.

### Clinical Implications and Limitations

Our findings sit within studies of parental mentalizing during middle childhood, thus revealing several theoretical and clinical implications. Firstly, this is one of the few studies (see also [26,99]) that has addressed parental insightfulness in middle childhood. Secondly, this study examined parental mentalizing in both parents throughout a multi-method approach with advantages in terms of a better understanding of the construct and opening up the possibility to differentiated clinical interventions based on the individual mentalizing dimensions involved.

Future research is needed, but the results suggest that parental mentalizing may play a different role based on the actors involved—mothers or fathers—and the specific mentalizing dimensions involved (e.g., [44]). Indeed, although many of the proposed variables expressed significant associations, the strongest relations are represented by parental pre-mentalizing modes similar to what happened in other studies that used the PRFQ [65] and maternal Insightfulness. Regarding fathers’ involvement, studies still maintain an important gap on both PRF and PI constructs, while, as Cowan and Cowan [100] suggested, it is important to bring him back into attachment research by studying his role in relation to the entire family system.

Finally, a last implication of this study relates to the importance that parental mentalizing has within dyadic parent–child interactions and related mentalization-based interventions to strengthen its positive influence also considering the increased effectiveness that parenting interventions may have in families with children during middle childhood (for a review [101]). More specifically, the construct of PI included a live approach—i.e., IA procedure—to measuring parental mentalizing, paving the way for clinical interventions that may use the same framework as video-feedback in order to improve a parent’s ability to use a reflective approach in their relationship with the child (e.g., [26,102]). Indeed, this measurement approach has the advantage of allowing a direct observation of actual functioning of parental mentalizing processes, being able to be used also in the evaluation of attachment-based interventions focused on improving the parent–child relationship (e.g., CONNECT program [103,104]; Circle of Security protocol [105]). In this regard, Oppenheim and colleagues [11] have shown an improvement in child’s internalizing and externalizing symptoms after the mother’s participation in a therapeutic intervention dedicated to increasing her levels of PI. Finally, moment-by-moment interaction is the main feature of IA allowing for its potential clinical application within video-feedback interventions (e.g., Video Intervention Therapy, VIT [106,107]; Video-feedback Intervention to promote Positive Parenting and Sensitive Discipline, VIPP-SD [108]) by helping parents explore new ways of constructing meanings within their parental representations and dyadic relationship with their child.

However, this study has several limitations. Firstly, the limited sample size and the subsequent statistical power reduction may have affected our results. Secondly, the cross-sectional design does not allow for causal inferences and requires future longitudinal studies, also expanding the exploration with the inclusion of clinical samples confirming our preliminary findings. It is also possible that a bias may have occurred in the sample selection, as we used a non-probabilistic sampling. Furthermore, concerning the adopted measures, one limitation is having used parent-report questionnaires to measure a child’s emotional–behavioral difficulties and social–emotional competencies. A recent study by An and Kochanska [66] showed a moderating effect of the mother’s pre-mentalizing modes so that the observed difficulties of the child were positively associated with the mother-report child’s difficulties only in mothers with low levels of pre-mentalizing modes. Therefore, considering the possibility that the presence of parental mentalistic deficits may affect parent-report child difficulties, it should be important to emphasize in future studies the use of a multi-method measurement approach that incorporates multiple measurements of a child’s emotional–behavioral difficulties and social–emotional competencies.

Despite the significant limitations, we believe that this work, which presents the first data on the link between maternal and paternal Insightfulness and child’s psychological functioning during middle childhood, proposes important implications related to the use of the Insightfulness Assessment procedure as a fundamental tool for the study of parental mentalizing processes and also for possible clinical applications.

## 5. Conclusions

The primary purpose of this study was to explore middle childhood using PI [14] and PRF [15] as key variables with respect to the child’s psychological functioning. Our findings are in line with previous literature showing a link between parental mentalizing, child’s emotional–behavioral difficulties and social–emotional competencies, thus emphasizing the importance of considering the parental ability to see their child’s mental states. These results further underline the importance of studying a child’s social–emotional competencies during middle childhood where these competencies assume greater relevance for the increasing relevance that the social context outside the family assumes for the child. Overall, these findings show the importance of considering parental mentalizing and underline the need to use a multi-method approach that overcomes the limitations deriving from a single measurement as occurred in this study about parent-report questionnaires. In addition, a stronger contribution of maternal than paternal mentalizing should be noted; considering the lack of research, these findings should be further explored in future studies. Finally, as recently argued by Luyten and colleagues [109], the limitation should be considered related to the sole use of mentalizing in the understanding of psychological functioning, emphasizing the need to expand the mentalizing framework by including it within a broader theoretical-clinical perspective. Following the direction proposed by these authors [109,110], the construct of mentalization could be integrated within an explanatory model that highlights the component of epistemic trust as a key element for positive psychological functioning. Based on these premises, future research may study mentalizing and epistemic trust by exploring their associations in specific stages of child development, including middle childhood, given the role that parental mentalizing may exert on the child’s epistemic trust as a result of more or less mentalistic interactions within the family system.

## Figures and Tables

**Table 3:** Correlations between parental mentalizing, child’s psychological symptoms and child’s social–emotional competencies.

	CBCL-I (Mother)	CBCL-E (Mother)	CBCL-T (Mother)	CBCL-I (Father)	CBCL-E (Father)	CBCL-T (Father)	SEC (Mother)	SEC (Father)
ITS (mother)	−0.36 *	−0.23	−0.36 *	−0.24	−0.24	−0.25	0.20	0.40 *
ITS (father) ^1^	−0.26	−0.10	−0.03	−0.16	−0.19	−0.06	0.35	0.27
PRFQ-PM (mother)	0.40 **	0.35 *	0.49 ***	0.22	0.31	0.34 *	−0.50 ***	−0.35 *
PRFQ-IC (mother)	0.03	−0.07	−0.01	0.29	0.24	0.21	0.05	0.10
PRFQ-CMS (mother)	−0.02	−0.20	−0.09	−0.02	−0.05	0.01	0.35 *	0.27
PRFQ-PM (father)	0.29	0.10	0.24	0.37 *	0.28	0.30	0.17	−0.11
PRFQ-IC (father)	−0.16	0.05	−0.05	−0.02	−0.04	−0.09	−0.05	0.46 **
PRFQ-CMS (father)	−0.10	0.12	0.02	−0.04	0.18	0.12	−0.37 *	0.01

Note. CBCL-I = Internalizing Symptoms; CBCL-E = Externalizing Symptoms; CBCL-T = Total Symptoms; SEC = Social Emotional Composite; ITS = Insightfulness Total Score; PRFQ-PM = Pre-Mentalizing Subscale; PRFQ-IC = Interest and Curiosity Subscale; PRFQ-CMS = Certainty About Mental States Subscale; ^1^ rho Spearman; * *p* < 0.05, ** *p* < 0.01, *** *p* < 0.001.

## Data Availability

Data available on request due to privacy and ethical restrictions.
